# Socio-Demographic Factors Influencing Monkeypox Vaccination Intentions Among Healthcare Workers and the General Population of Makurdi, Nigeria: A Cross-Sectional Study

**DOI:** 10.7759/cureus.71828

**Published:** 2024-10-19

**Authors:** Adewale Lawrence

**Affiliations:** 1 Pharmaceutical Medicine, Bioluminux Clinical Research, Naperville, USA

**Keywords:** healthcare workers, monkeypox, nigeria, public health, socio-demographic factors, vaccination intentions

## Abstract

Background

Monkeypox, now known as mpox, is a viral zoonotic disease caused by the monkeypox virus (MPXV) and is endemic to parts of Central and West Africa. Historically considered a regional health issue, mpox has gained global attention due to recent outbreaks in non-endemic regions. In 2022, the World Health Organization (WHO) declared mpox a Public Health Emergency of International Concern (PHEIC) following widespread transmission in countries where the virus had not been previously reported. This declaration was renewed in August 2024 after a surge in cases, particularly across Africa, including the Democratic Republic of Congo (DRC) and neighboring countries, where a more transmissible strain (Clade 1b) has rapidly spread, complicating efforts to control its transmission.

Objective

This study evaluated the socio-demographic factors that influenced monkeypox vaccination intentions among healthcare workers and the general population in Benue State, Nigeria. The findings provide insights into key determinants of vaccine hesitancy and acceptance, aimed at informing future public health interventions to improve vaccine uptake.

Methodology

This cross-sectional study, conducted from July to September 2024 in Makurdi, Benue State, Nigeria, evaluated socio-demographic factors influencing monkeypox vaccination intentions among healthcare workers (HCWs) and high-risk populations. The study, prompted by the WHO's declaration of monkeypox as a PHEIC, surveyed 377+ participants including HCWs, people living with HIV, and men who have sex with men.

Results

Key findings revealed significant differences in vaccination intentions based on marital status (p-value = 0.02), with no significant variations by gender or age. COVID-19 vaccination status was associated with monkeypox vaccination intentions. The 30-60 age group demonstrated the highest monkeypox awareness (75.2%). Healthcare workers showed high vaccination intent (83%) and were identified as the most reliable information source (p-value = 0.03). Higher education levels correlated with greater vaccine trust (p-value = 0.003), while lower education was linked to reduced awareness and increased stigma perceptions.

Conclusion

The study recommends enhancing HCW training, addressing socioeconomic barriers to vaccination, implementing targeted education campaigns, and focusing on trust-building strategies. Limitations include the study's urban focus and potential language barriers. In conclusion, socio-demographic factors significantly influence monkeypox vaccination intentions, underscoring the need for targeted interventions and improved vaccine access to mitigate the impact of monkeypox in Nigeria and affected regions.

## Introduction

Monkeypox, a viral zoonotic disease caused by the monkeypox virus (MPXV), has returned as a major public health concern, especially in West and Central Africa [1]. Since the first documented case in 1970, Nigeria has experienced repeated outbreaks [2]. The virus is prevalent in numerous parts of Nigeria, and the rising number of cases highlights the critical need for comprehensive public health initiatives, notably immunization, to restrict its spread [3]. Vaccination is a vital technique for combating infectious diseases, including monkeypox [4]. In light of the recent outbreaks, the Nigerian government and international health organizations have emphasized the development and distribution of monkeypox vaccinations [5]. However, the effectiveness of these vaccination efforts is greatly dependent on the willingness of both healthcare workers (HCWs) [6] and the general public to accept the vaccine. HCWs are especially influential because their attitudes and behaviors can considerably influence public opinion and acceptance of immunization initiatives [5].

Despite the widely accepted importance of vaccination, hesitation remains a significant hurdle [7]. Several socio-demographic characteristics, such as age, gender, education, socioeconomic level, and cultural attitudes [8], can have a substantial impact on people's intentions to get vaccinated. Younger people may be more amenable to vaccination than older generations, who may have a history of mistrusting medical interventions. Furthermore, healthcare providers' knowledge and attitudes concerning monkeypox and its vaccine can either boost or undermine public confidence [9]. According to research, Nigeria's socioeconomic status of the population influences health behaviors significantly [10]. Areas with lower educational attainment and limited access to healthcare resources frequently have greater rates of vaccine reluctance. Furthermore, cultural factors, such as traditional beliefs and stigmas associated with specific diseases, can discourage people from obtaining vaccination [11]. The interaction of these elements generates a complex environment that demands a thorough investigation to better understand the motivations and challenges influencing vaccination intentions [12].

In Benue State, where monkeypox cases have been documented, knowing the dynamics of vaccination intentions is especially important. The state's different demographic characteristics allow researchers to investigate how many factors influence people's willingness to vaccinate against monkeypox [13]. Benue State, in Nigeria's north-central area, is home to an estimated five million people, the majority of whom are Tiv, Idoma, and Igede ethnic groups. The population is predominantly youthful, with approximately 44% under the age of 15 and a median age of 18 years [14]. Socioeconomic conditions are difficult, with around 60% of the population living below the national poverty line and a literacy rate of around 57%, with considerable differences between urban and rural areas. The state's economy is predominantly agrarian, employing more than 70% of the population [15], yet substantial unemployment rates continue, particularly among young people. Access to healthcare is limited, with a doctor-to-population ratio of approximately 1 to 5,000, resulting in insufficient service delivery, particularly in rural areas [7]. Understanding the socio-demographic characteristics that influence vaccination intentions among healthcare personnel and the general public is critical for increasing vaccine uptake and community engagement in treating infectious illnesses such as monkeypox.

The recurrence of monkeypox in Nigeria, especially during the 2022 outbreak, emphasizes the critical need for effective immunization strategies. Understanding the factors that influence vaccination intentions is critical for establishing targeted treatments, particularly in locations such as Benue State, where transmission rates are increasing. HCWs play an important role in public health outcomes; their knowledge and attitudes can influence community opinions about vaccination. Age, gender, education level, and cultural attitudes are all common sociodemographic characteristics that influence people's intentions to get immunizations. By investigating these aspects, this project hopes to uncover specific barriers and facilitators to vaccination, thereby reducing vaccine hesitancy caused by disinformation and historical mistrust. The findings will enhance public health planning and policy development, allowing health officials to conduct successful educational efforts that resonate given the region's specific sociodemographic setting. Finally, this research will help to increase vaccination rates and improve health outcomes for monkeypox and other infectious illnesses, giving valuable insights for global health efforts.

Objective

This study evaluated the socio-demographic factors that influenced monkeypox vaccination intentions among HCWs and the general population in Benue State, Nigeria. The findings provide insights into key determinants of vaccine hesitancy and acceptance, aimed at informing future public health interventions to improve vaccine uptake.

## Materials and methods

Study design and study setting

This cross-sectional survey was designed to assess the socio-demographic factors influencing monkeypox vaccination intentions among HCWs and the general population in Benue State, Nigeria. The study was conducted in Makurdi, the capital of Benue State, known for its diverse urban population and healthcare infrastructure. The Federal Medical Center, Makurdi, served as one of the primary data collection sites, supplemented by outreach efforts to various community organizations and high-risk populations, such as people living with HIV (PLHIV) and men who have sex with men (MSM). These groups were included due to their higher vulnerability and risk for monkeypox transmission, reflecting the importance of understanding their vaccination intentions.

Study population

The study population included HCWs at the Federal Medical Center in Makurdi and individuals from high-risk groups within the Makurdi metropolis, particularly PLHIV and MSM. These groups were selected due to their increased vulnerability to monkeypox infection and the critical need for targeted vaccination interventions. In addition, samples were drawn from the general population within Makurdi to provide a more comprehensive socio-demographic analysis of vaccination intentions across different community segments.

Sample size calculation

**Figure 1:**
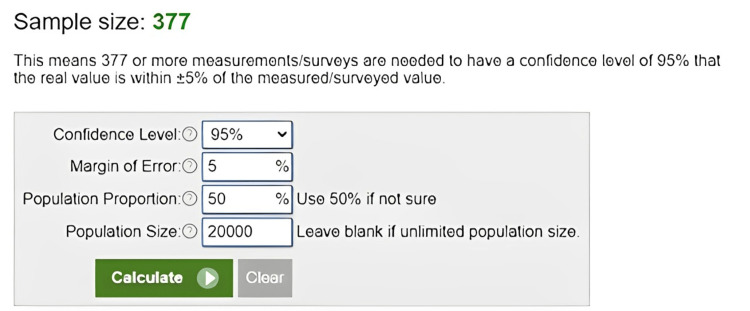
Sample size calculation details

The sample size was calculated using Raosoft software (Raosoft, Inc., Seattle, United States). This means 377 or more measurements/surveys are needed to have a confidence level of 95% that the real value is within ±5% of the measured/surveyed value. The details are mentioned in Figure 1.

Sample size discrepancy

Based on the data provided, while the calculated sample size for the study was at least 377 participants, the minor discrepancy between the calculated and actual sample sizes should be noted. The implications of this difference on the study's statistical power are likely minimal, given that the sample is still close to the target size. However, the authors should acknowledge this small deviation and explain that although the sample size was slightly lower than anticipated, it is unlikely to substantially affect the generalizability of the results or the study's ability to detect statistically significant relationships, as the difference is marginal.

Sampling technique

A snowball sampling technique was employed for participant recruitment. Initial participants were enrolled at the Federal Medical Center and designated MSM clinics within Makurdi. Following this, the survey link was disseminated via social media platforms, including WhatsApp, Facebook, and Telegram, to reach a broader and more diverse audience. Participants who consented to take part in the study were provided with either a paper-based or online self-administered questionnaire, depending on their preference.

Eligibility criteria

Inclusion Criteria

Participants included adults aged 18 years and older, specifically HIV-positive individuals, MSMs, and HCWs at the Federal Medical Center in Makurdi. MSM individuals identified within the Makurdi metropolis who consented to participate were also included.

Exclusion Criteria

Individuals excluded from the study were those under 18 years of age, HIV-negative persons, and those from high-risk groups (MSM and HCWs) who declined or were unable to provide consent due to ill health. The rationale behind the exclusion criteria is as follows:

Age restriction: The exclusion of minors is likely due to ethical considerations and the need for parental consent.

HIV status: The focus on HIV-positive individuals is probably because they represent a high-risk group for monkeypox. However, this should be explicitly stated and justified.

Consent issues: Exclusion based on the inability to provide consent is a standard ethical practice, but the specific mention of ill health suggests a need for further explanation. The authors should clarify if there were particular health conditions that might affect the ability to participate or influence vaccination intentions.

Data collection procedure

This cross-sectional study assessed the knowledge, attitudes, and acceptability of monkeypox vaccination strategies among high-risk populations, including PLHIV, MSM, and HCWs in Benue State, North Central Nigeria. Participant enrollment was conducted at the Federal Medical Centre Makurdi and designated MSM clinics in the Makurdi metropolis from July to September 2024.

Data were collected using a self-administered questionnaire in English [16], which was divided into three sections. The questionnaire, previously tested and validated, included:

Section One

Section one collected socio-demographic data such as age, gender, nationality, marital status, education level, occupation, financial status, and the presence of chronic illness.

Section Two

Section two assessed COVID-19 vaccine uptake, exposure to MPOX, knowledge of MPOX-related fatalities, and intentions to receive the MPOX vaccine.

Section Three

Section three utilized the 5C scale to evaluate psychological antecedents to vaccination, consisting of 15 questions spread across five subscales, with responses recorded on a seven-point Likert scale.

The questionnaire was piloted, with participants completing it in 5-7 minutes, and demonstrated strong internal consistency (Cronbach’s alpha > 0.8).

To ensure inclusivity, both online and hard-copy versions of the questionnaire were made available. The online survey was hosted on platforms such as Google Forms, while hard copies were distributed through community hospitals, healthcare facilities, and local support groups for participants without reliable internet access. Trained personnel facilitated the distribution and collection of hard-copy questionnaires. Data from the hard copies were manually entered into the online platform to ensure a consolidated and robust dataset for analysis.

Statistical analysis

The statistical analysis plan involved checking the data for completeness, accuracy, and consistency prior to analysis.

Data preparation

Checking Data Completeness, Accuracy, and Consistency

The data was reviewed to ensure that all responses were complete and consistent across variables. Frequency tables and cross-tabulations were used to check for inconsistencies (e.g., ensuring gender and age groups aligned with expectations). Outliers were identified and corrected if necessary.

Addressing Missing Values

Missing values were handled by listwise deletion, as the amount of missing data was minimal (only 1.6% of education data). This approach was appropriate due to the small percentage, and it allowed for maintaining the integrity of the remaining data.

Descriptive Statistics

Measures of central tendency and dispersion: For categorical variables (e.g., gender, marital status, occupation), frequency distributions and percentages were used to describe the data.

For continuous variables (e.g., age), the mean and standard deviation were calculated to represent central tendency and dispersion.

Inferential Statistics

Chi-square tests: The chi-square test was applied to analyze categorical variables, such as gender, marital status, nationality, and vaccination intentions. For example, chi-square was used to assess differences in monkeypox vaccination intentions by gender (p-value = 0.6) and marital status (p-value = 0.02).

Assumptions

The assumptions for the chi-square tests were checked by ensuring that the expected frequencies in each cell were adequate (i.e., no more than 20% of cells had expected counts less than 5).

Software and Significance Level

Software: The data analysis was conducted using IBM SPSS Statistics for Windows, Version 25 (Released 2017; IBM Corp., Armonk, New York, United States).

Significance level: A significance level (α) of 0.05 was used for all inferential tests. This means that results with a p-value less than 0.05 were considered statistically significant, indicating that there is less than a 5% probability that the observed associations occurred by chance.

Ethical consideration

This research adhered to the principles outlined in the Declaration of Helsinki and was conducted with approval from the Health Research Ethics Committee, with reference number FMH/FMC/HREC/108/VOL.I. Informed consent was obtained from all participants before their involvement in the study. Participation was voluntary, and participants had the right to withdraw at any time without consequence. Confidentiality was assured, and participants’ data were anonymized to protect their privacy. The study was conducted with integrity, transparency, and full respect for participants' rights and welfare. Efforts were made to minimize any potential risks, and the benefits of the research were ensured to outweigh any possible harms. All conflicts of interest were disclosed, and the research adhered to relevant laws, regulations, and ethical standards throughout its duration.

## Results

Demographic characteristics of the participants

Table 1 summarizes the demographic features of the study's respondents. In terms of gender, the majority were females (63.9%), with males making up 35.8% and a small proportion identifying as "others" (0.3%). In terms of age, the majority of respondents (74.8%) were between the ages of 30 and 60, with 20.4% falling between the ages of 18 and 29, and 4.8% being older than 60. Almost all participants (99.7%) were Nigerian, with only 0.3% claiming another nationality. In terms of marital status, the majority (62.1%) were married, with 22.5% single, 12.2% widowed, 2.7% having a companion, and 0.5% identifying as "other." In terms of where they lived, 68.2% lived in cities or towns, 31% in rural or village settings, and only 0.3% reported living somewhere else. Regionally, the majority of responders (65.8%) came from Benue State, with 32.6% from other regions. According to occupation, 43.5% were physicians or physician assistants, 16.4% were nurses or midwives, 5.3% were pharmacists or pharmacy technicians, and 34.5% were laboratory technicians. In terms of education, 53.8% had no formal education, 44.6% had completed pre-college or high school, and 1.6% of data was missing.

**Table 1:** Demographic characteristics of the participants

Variables	Category	Frequency (n = 377)	Percentage 100%
Gender	Male	135	35.8
	Female	241	63.9
	Others	1	0.3
Age	18-29 years	77	20.4
	30-60 years	282	74.8
	Above 60 years	18	4.8
Nationality	Nigerian	376	99.7
	Other	1	0.3
Marital status	Single	85	22.5
	Married	234	62.1
	Having partner	10	2.7
	Widow	46	12.2
	Other	2	0.5
Living area	Urban/city	257	68.2
	Rural/village	117	31.0
	Other	1	0.3
Region	Benue state	250	65.8
	Other	126	32.6
Occupation	Physician/physician assistant	164	43.5
	Nurse/midwife	62	16.4
	Pharmacist/pharmacy technician	20	5.3
	Laboratory technician	130	34.5
Highest level of education/degree you have completed	Not educated	203	53.8
	Pre-college/high school	168	44.6

Cross tabulation of socio-demographic factors and intention to get monkeypox vaccination

Table 2 explains the data sheds light on respondents' plans to obtain the monkeypox vaccination based on their demographic features. Regarding gender, 66 of 132 male respondents expect to get vaccinated, while 66 do not. About 111 of the 238 female respondents plan to get vaccinated, while 127 do not. The sole respondent who identified as "others" does not intend to get vaccinated. The difference in vaccination intentions across genders is not statistically significant (p-value = 0.6). Regarding age, the majority of respondents aged 18-29 (32 out of 75) expect to get vaccinated, while 43 do not. In the 30-60 age group, 136 respondents want to get vaccinated, while the remaining 142 do not, revealing no significant age-related differences (p-value = 0.2). Regarding nationality, 178 Nigerian respondents plan to get vaccinated, while 193 do not. There is no significant difference across nationalities (p-value = 0.7). When it comes to marital status, the majority of married respondents (96%) want to get vaccinated, followed by singles (85) and widows (46). The p-value of 0.02 indicates a statistically significant variation in vaccination intentions depending on marital status.

**Table 2:** Monkeypox vaccination intentions by demographic characteristics * indicates statistical significance (p-value < 0.05)

Gender									
		Male		Female		Others		Total (n = 377)	p-value
Monkeypox vaccination	Yes	66		111		1		178	0.6
	No	66		127		0		193	
	Others	3		3		0		6	
Age									
		18-29 years		30-60 years		Above 60 years			
Do you intend to get a monkeypox vaccination	Yes	32		136		10		178	0.15
	No	43		142		8		193	
	Others	2		4		0		6	
Nationality									
		Nigerian				Others			
Do you intend to get a monkeypox vaccination	Yes	178				0		178	0.7
	No	193				0		193	
	Others	6				0		6	
Marital status									
		Single	Married		Having partner	Widow	Other		
Do you intend to get a monkeypox vaccination	yes	26	96		1	25	1	149	0.02*
	no	43	110		7	15	1	176	
	others	10	24		2	5	0	41	

Cross-tabulation of COVID-19 vaccination status and monkeypox vaccination intention

Table 3 explains the association between age, COVID-19 vaccination status, and monkeypox infection. For COVID-19 immunization, 14 people aged 18 to 29 are fully vaccinated, 57 have taken the first dose and plan to take the second, and five have taken the first dosage but do not intend to finish the vaccination. In the 30-60 age range, 44 are fully vaccinated, 222 intend to receive the second dose, and 13 do not plan to do so. Among those over 60, one is fully vaccinated, 15 aim to complete their vaccination, and two are not. The p-value of 0.3 suggests no significant difference in COVID-19 immunization status between age groups. Regarding monkeypox infection, nine respondents aged 18-29 reported being infected, compared to 62 who were not and six who were unclear. In the 30-60 age bracket, eight people reported infection, 239 were not infected, and 34 were doubtful. Among individuals over 60, one has had monkeypox, 16 have not, and one is unsure. The p-value is 0.000, indicating a highly significant difference in monkeypox infection rates between age groups. When asked if they knew someone who had died from monkeypox, 51 respondents aged 18 to 29 indicated "yes," whereas 26 said "no." In the 30-60 age range, 196 knew someone who had died, 85 did not, and one was unclear. Among those over 60, 14 knew someone who died and four did not. The p-value for this is 0.3, indicating that there are no significant differences in knowledge of monkeypox-related mortality among age groups.

**Table 3:** Perception of vaccination and intention to get monkeypox vaccination * indicates statistical significance (p-value < 0.05)

				Did you receive COVID-19 vaccine			Total	p-value
				Fully vaccinated	Took first dose, going to take second	Took first dose, will not take second		
Age	18-29 years			14	57	5	76	0.3
	30-60 years			44	222	13	279	
	Above 60 years			1	15	2	18	
				Have you had monkeypox infection			-	0.000*
Age				Yes	No	I don't know		
		18-29 years		9	62	6	77	
		30-60 years		8	239	34	281	
		Above 60 years		1	16	1	18	
				Do you know anyone who died due to monkeypox, that you know?				0.3
				Yes	No	I don't know		
Age			18-29 years	51	26	0	77	
			30-60 years	196	85	1	282	
			Above 60 years	14	4	0	18	

Cross-tabulation of confidence in vaccine safety and monkeypox vaccination intention

Table 4 explains the correlation between respondents' attitudes toward vaccinations and their intention to receive the monkeypox vaccine. It found that those who intend to get vaccinated strongly agree with the effectiveness of vaccines, while those not intending to vaccinate are split between neutral and strongly disagreeing. The belief in vaccine-preventable diseases is also significant, with 40 of those intending to get vaccinated strongly agreeing. The belief that vaccine-preventable diseases are not severe enough to warrant vaccination is also significant. The study also found a strong connection between weighing risks and benefits and the intention to vaccinate.

**Table 4:** Cross-tabulation of confidence in vaccine safety and monkeypox vaccination intention * indicates statistical significance (p-value < 0.05)

Vaccinations are effective											
Do you intend to get a monkeypox vaccination				Category	Strongly agree	Agree	Neutral	Disagree	Strongly disagree	Total	p-value
				Yes	107	48	16	5	2	178	0.000*
				No	28	146	12	5	2	193	
				Others	0	1	4	1	0	6	
Total					135	135	195	32	11	377	
Vaccination is unnecessary because vaccine-preventable diseases are not common anymore											
Do you intend to get a monkeypox vaccination	Category				Strongly agree	Agree	Neutral	Disagree	Strongly disagree		
	Yes				48	54	18	34	23	177	0.000*
	No				20	91	16	56	10	193	
	Others				1	3	1	0	1	6	
Total					69	69	148	35	90	376	
Vaccine-preventable diseases are not so severe that I should get vaccinated											
Do you intend to get a monkeypox vaccination		Category			Strongly agree	Agree	Neutral	Disagree	Strongly disagree		
		Yes			12	21	8	94	41	176	0.000*
		No			3	39	11	115	25	193	
		Others			0	1	2	3	0	6	
Total					15	15	61	21	66	375	
When I think about getting vaccinated, I weigh the benefits and risks to make the best decision possible											
Do you intend to get a monkeypox vaccination			Category		Strongly agree	Agree	Neutral	Disagree	Strongly disagree		
			Yes		75	85	4	8	6	178	0.000*
			No		48	126	6	9	4	193	
			Others		1	2	3	0	0	6	
Total					124	213	13	17	10	377	
It is important for me to fully understand the topic of vaccination before I get vaccinated											
Do you intend to get a monkeypox vaccination		Category			Strongly agree	Agree	Neutral	Disagree	Strongly disagree		
		Yes			18	17	2	71	70	178	0.000*
		No			4	47	9	97	35	192	
		Others			0	2	3	1	0	6	
Total					22	66	14	169	105	376	
Vaccination is a collective action to prevent the spread of disease											
Do you intend to get a monkeypox vaccination	Category				Strongly agree	Agree	Neural	Disagree	Strongly disagree		
	Yes				118	43	3	6	7	177	0.000*
	No				63	121	2	1	4	191	
	Others				2	4	0	0	0	6	
Total					183	168	5	7	11	374	

## Discussion

The increasing incidence of monkeypox in Nigeria, particularly among high-risk populations such as PLHIV, MSM, and HCWs, underscores the critical need to assess vaccination knowledge, attitudes, and acceptability in these groups. This study evaluated factors influencing monkeypox vaccination intentions among HCWs and the general population in Benue State, Nigeria. The findings demonstrate that socio-demographic characteristics significantly impact vaccination intentions, particularly gender, age, and education level.

Contrary to our initial interpretation, the study found no statistically significant difference in vaccination intentions between genders (p-value = 0.6). This finding challenges the assumption that women are generally more likely to engage in health-seeking behaviors, at least in the context of monkeypox vaccination in this population. It's important to note that factors influencing vaccination intentions may be more complex than gender alone and could involve intersections with other socio-demographic variables not fully explored in this study. This finding is consistent with previous research demonstrating that women are generally more engaged in health-seeking behaviors and have higher health literacy, contributing to increased vaccination intent [17]. The gender disparity in vaccination participation is a complex issue that requires further investigation, especially in patriarchal societies where public health efforts need to encourage equal participation [18].

The study found that people aged 30 to 60 had the highest levels of monkeypox awareness (75.2%); however, there were no significant differences across age groups (p-value = 0.2). This uniformity in intentions across age groups is intriguing and warrants further investigation. It may suggest that public health messaging about monkeypox has been relatively consistent across age groups, or that other factors are more influential in shaping vaccination intentions [19].

Prior studies have emphasized the importance of inclusive health education campaigns to engage all age groups and raise disease awareness. The lack of significant age-related differences may suggest that public health programs have been relatively successful in reaching diverse age groups, though gaps may persist in specific subpopulations.

HCWs demonstrated a high level of awareness about monkeypox vaccinations, with 83% expressing intent to vaccinate. This finding is encouraging, as HCWs play a crucial role in promoting immunization. However, it's important to note that this study did not directly measure HCWs' skepticism about vaccine safety and efficacy. The high intention to vaccinate suggests general acceptance but doesn't preclude the existence of concerns or hesitancies that weren't captured by our measures [20]. Addressing these concerns is vital, as vaccine reluctance among HCWs could hinder broader public health campaigns [21]. Future research should explore the nuances of HCWs' attitudes toward the monkeypox vaccine, including any reservations they might have.

The study revealed a significant association between education levels and the vaccine trust indicator (p-value = 0.003), suggesting that individuals with higher educational attainment are more likely to trust vaccines. This finding aligns with existing literature on health literacy and vaccine perceptions [22]. However, it's crucial to consider that education level may be a proxy for other socioeconomic factors that influence vaccine trust and access to health information.

Additionally, HCWs were identified as the most reliable sources of information about monkeypox (p-value = 0.03) reinforcing their critical role in public health education. This finding is consistent with earlier research [23], which highlights the influence of HCWs on patient behavior and vaccination decisions. HCWs must be equipped to counteract misinformation on social media and enhance their communication skills. Despite widespread awareness, some HCWs remain skeptical about vaccine safety and efficacy, which poses a serious challenge as their reservations can erode public trust in vaccination campaigns [24]. Another significant finding was the relationship between education and perceptions of monkeypox-related stigma. More educated individuals reported lower perceptions of stigma (p-value = 0.03), consistent with previous studies [25]. Education and public health campaigns can play a key role in reducing stigma, improving health literacy, and overcoming healthcare barriers, particularly for marginalized groups, by addressing socio-demographic factors and implementing targeted interventions.

Key findings and their implications

The study revealed that marital status significantly influenced vaccination intentions (p-value = 0.02). This finding suggests that family dynamics and social structures play a crucial role in health decision-making processes. Public health campaigns should consider these familial influences when designing vaccination promotion strategies. Contrary to initial expectations, gender and age did not show significant associations with vaccination intentions. This challenges some preconceived notions about health-seeking behaviors and emphasizes the need for a nuanced understanding of demographic factors in the context of monkeypox vaccination.

The 30-60 age group demonstrated the highest level of monkeypox awareness (75.2%). This finding identifies a key demographic that may serve as potential advocates for vaccination efforts. However, it also highlights the need for increased awareness campaigns targeting younger and older age groups. Education levels were strongly associated with vaccine trust (p-value = 0.003), underscoring the critical role of health literacy in shaping perceptions of vaccine safety and efficacy. This emphasizes the need for tailored educational interventions that address varying levels of health literacy across the population.

The high intention to vaccinate among HCWs (83%) is encouraging, as they play a pivotal role in influencing public opinion and promoting vaccination. However, the study also revealed the need for continued education and support for this group to address any underlying concerns or misconceptions they may have about the vaccine.

The study identified significant knowledge gaps and stigma perceptions, particularly among those with lower educational attainment. This highlights the need for accessible, culturally sensitive health communication strategies that can effectively reach and engage diverse population segments.

The observed association between COVID-19 vaccination status and monkeypox vaccination intentions suggests potential carry-over effects in vaccine acceptance. This finding could inform strategies that leverage existing vaccine infrastructure and public health messaging to promote monkeypox vaccination.

Recommendations

*HCW*​​​​​​* Training*

HCW training provides comprehensive education and training for HCWs to enhance their understanding of the monkeypox vaccine and its importance.

Address Socioeconomic Barriers

Address socioeconomic barriers and develop strategies to reduce socioeconomic barriers to vaccination, particularly in rural areas where access to healthcare is limited. This may include mobile clinics, subsidized vaccination programs, and integrating vaccination with other routine healthcare services.

Further Research

Additional studies need to be conducted to explore other potential factors influencing vaccination intentions, such as religious beliefs, traditional health practices, and misinformation, to create a more comprehensive understanding of vaccine hesitancy in the region.

Limitations and unexpected findings

Several limitations and unexpected findings deserve attention, such as the lack of significant gender differences in vaccination intentions, which contradicts some previous research and highlights the need for a more nuanced exploration of gender's role in health decision-making in this context. While we found high vaccination intentions among HCWs, we lack data on their specific concerns or reservations. This gap in our understanding could be addressed in future studies.

The study's focus on urban areas and specific high-risk groups may limit the generalizability of findings to rural populations or other demographic groups. The relationship between COVID-19 vaccination status and monkeypox vaccination intentions, while noted, requires more detailed analysis to understand the nature and strength of this association. The impact of stigma on vaccination intentions, particularly among less educated individuals, was observed but not thoroughly explored. This represents an important area for future research.

## Conclusions

This study on monkeypox vaccination intentions in Benue State, Nigeria, provides a crucial foundation for understanding the complex factors influencing vaccine acceptance in endemic regions. The findings underscore the need for a multifaceted approach to promoting monkeypox vaccination, one that considers the diverse socio-demographic landscape and addresses both individual and community-level factors. By implementing targeted interventions, improving vaccine access, and building public trust, there is significant potential to increase vaccination coverage and mitigate the impact of monkeypox in Benue State and similar regions. The insights gained from this study can inform the development of evidence-based strategies to combat the spread of monkeypox and improve overall vaccination rates in Nigeria and beyond.

As the global health community continues to grapple with emerging and re-emerging infectious diseases, studies like this provide valuable guidance for creating responsive, effective, and equitable public health interventions. The lessons learned here can contribute not only to addressing monkeypox but also to strengthening overall vaccine acceptance and public health resilience in the face of future health challenges.

## Appendices

Questionnaire 1

Monkeypox is a disease spreading worldwide, and we, doctors and researchers from Bioluminux Clinical United States and the Federal University of Health Sciences Otukpo, Benue State, Nigeria, invite you to participate in this study. The study aims to assess the psychological motives for vaccinations against monkeypox.

Consent question:

1.     Participate in the survey

a.     Agree

b.     Disagree

Section 1: Socio-demographics questions:

2.     Gender

a.     Male

b.     Female

c.     Other

3.     Age in years ………….

4.     Nationality …………..

5.     Country where you are living now: ………….

6.     Marital status:

a.     Single

b.     Married

c.     Having partner

d.     Widow

e.     Other

7.     Living area:

a.     Urban/City

b.     Rural/Village

c.     Other: …………

8.     Region:

a.     Benue State

b.     Other:…………….

9.     Financial status:

a)     Low income

b)    Middle income

c)     Upper income

10.  What is the highest level of education/degree you have completed?

a)     Not educated

b)    Pre-college/High school

c)     Professional/Technical

d)    Bachelor

e)     Diploma

f)     Postgraduate (Master)

g)    Postgraduate (PhD)

11.  Do you suffer from any chronic diseases?

a)     Yes

b)    No

12.  Occupation

a)     Physician /Physician Assistant

b)    Nurse/midwife

c)     Pharmacist/ pharmacy technician

d)    Laboratory technician

e)     Public health personnel

13.  Other field (not in the healthcare field)

a)     Student

b)    Unemployed

c)     Other……………………………………..

Section II

14.  Did you receive COVID-19 vaccine

a)     Fully vaccinated

b)    Took the first dose, going to take the second

c)     Took the first dose, and will not take the second

d)    Not going to take the vaccine

15.  Have you had a Monkeypox infection?

a)     Yes

b)    No

c)     I don't know

16.  Do you know anyone who died due to Monkeypox, that you know?

a)     Yes

b)    No

c)     I don't know

17.  Do you intend to get a Monkeypox vaccination?

a)     Yes

b)    No

Section III: Please answer these questions about Monkeypox vaccinations:

18.  I am completely confident that vaccines are safe

a)     Strongly agree

b)    agree

c)     neutral

d)    disagree

e)     strongly disagree

19.  Vaccinations are effective

a)     strongly agree

b)    agree

c)     neutral

d)    disagree

e)     strongly disagree

20.  Regarding vaccines, I am confident that public authorities decide in the best interest of the community

a)     strongly agree

b)    agree

c)     neutral

d)    disagree

e)     strongly disagree

21.  Vaccination is unnecessary because vaccine-preventable diseases are not common anymore

a)     strongly agree

b)    agree

c)     neutral

d)    disagree

e)     strongly disagree

22.  My immune system is so strong, that it also protects me against diseases

a)     strongly agree

b)    agree

c)     neutral

d)    disagree

e)     strongly disagree

23.  Vaccine-preventable diseases are not so severe that I should get vaccinated

a)     strongly agree

b)    agree

c)     neutral

d)    disagree

e)     strongly disagree

24.  Everyday stress prevents me from getting vaccinated

a)     strongly agree

b)    agree

c)     neutral

d)    disagree

e)     strongly disagree

25.  For me, it is inconvenient to receive vaccinations

a)     strongly agree

b)    agree

c)     neutral

d)    disagree

e)     strongly disagree

26.  Visiting the doctors makes me feel uncomfortable; this keeps me from getting vaccinated

a)     strongly agree

b)    agree

c)     neutral

d)    disagree

27.  When I think about getting vaccinated, I weigh the benefits and risks to make the best decision possible

a)     strongly agree

b)    agree

c)     neutral

d)    disagree

e)     strongly disagree

28.  For every vaccination, I closely consider whether it is useful for me

a)     strongly agree

b)    agree

c)     neutral

d)    disagree

e)     strongly disagree

29.  It is important for me to fully understand the topic of vaccination before I get vaccinated

a)     strongly agree

b)    agree

c)     neutral

d)    disagree

e)     strongly disagree

30.  When everyone is vaccinated, I don't have to get vaccinated too

a)     strongly agree

b)    agree

c)     neutral

d)    disagree

e)     strongly disagree

31.  I get vaccinated because I can also protect people with a weaker immune system

a)     strongly agree

b)    agree

c)     neutral

d)    disagree

e)     strongly disagree

32.  Vaccination is a collective action to prevent the spread of disease

a)     strongly agree

b)    agree

c)     neutral

d)    disagree

e)     strongly disagree
